# *Culex quinquefasciatus* (Diptera: Culicidae) From Florida Transmitted Zika Virus

**DOI:** 10.3389/fmicb.2018.00768

**Published:** 2018-04-26

**Authors:** Chelsea T. Smartt, Dongyoung Shin, Seokyoung Kang, Walter J. Tabachnick

**Affiliations:** ^1^Florida Medical Entomology Laboratory, Department of Entomology and Nematology, IFAS, University of Florida, Gainesville, FL, United States; ^2^Infectious Diseases & Immunology, College of Veterinary Medicine, University of Florida, Gainesville, FL, United States

**Keywords:** Zika virus, mosquito, *Culex quinquefasciatus*, transmission, saliva

## Abstract

We report a laboratory colony of *Culex quinquefasciatus* mosquitoes were experimentally able to salivate Zika virus (ZIKV, *Flaviviridae; Flavivirus*) at 16 days post infection (dpi). ZIKV RNA was detected in bodies and in saliva deposited on filter paper cards with subsequent studies demonstrating the presence of live ZIKV in saliva.

## Introduction

ZIKV is a single stranded RNA mosquito-borne arbovirus that can cycle between mosquitoes and humans in an urban environment. It is mainly transmitted to humans by *Aedes aegypti* (Duffy et al., 2009; Chouin-Carneiro et al., 2016; Ferreira-de-Brito et al., 2016; Guerbois et al., 2016). A Zika outbreak rapidly spread through the Americas including the southern United States in 2016 transmitted primarily by *Ae. aegypti* populations (Chouin-Carneiro et al., 2016). Field collected *Cx. quinquefasciatus* from Brazil were found infected with genome fragments of ZIKV and field collections from Mexico were found infected with live ZIKV (Guedes et al., 2017; Elizondo-Quiroga et al., 2018). However, results from many laboratory studies on the competence of *Culex* species for ZIKV are mixed (Amraoui et al., 2016; Boccolini et al., 2016; Guo et al., 2016; Huang et al., 2016; Kenney et al., 2017) leading to the conclusion that Culex are unlikely to play a role in ZIKV transmission (Roundy et al., 2017). Support for assessing *Culex* competence for ZIKV was provided by a recent modeling study that suggested 35 species of mosquitoes may be able to transmit ZIKV, including *Cx. quinquefasciatus* and *Cx. pipiens* from the United States (Evans et al., 2017). Knowledge about the competence for ZIKV in populations of *Cx. quinquefasciatus* is needed to assess risk and prevent ZIKV transmission in regions where this species is found.

Geographic variation in mosquito competence for arboviruses is well established (Tabachnick, 2013). Here we studied a Gainesville, Florida population of *Cx. quinquefasciatus*, shown to be highly competent for West Nile virus (WNV, *Flaviviridae; Flavivirus*) compared to a population collected in Vero Beach, Florida (Shin et al., 2014).

## Materials and methods

A laboratory colony of *Cx. quinquefasciatus* collected in 1995 (generation ≥ 100) were infected with a ZIKV strain (Asian lineage, Gen Bank KU501215.1) from Puerto Rico (collected December 2015, human serum; Lanciotti et al., 2016). Viral stocks were propagated in culture using African green monkey (Vero) cells following standard procedures and established methods (Faye et al., 2013). Four-day old female mosquitoes were exposed for 1 h at 28°C to freshly harvested ZIKV mixed with chicken blood in Alsevers (Hemostat, Dixon, CA; diluted 1:1) and 1% ATP (100 mM) on soaked cotton pledgets warmed to 35°C for 10 min. Following feeding, fully engorged females were transferred to screened cages and held at 28°C and 75% humidity. Mosquitoes were provided 20% sugar solution for the duration of the experiment. A sample of the blood meal and three freshly fed fully engorged mosquitoes were stored at −80°C to later characterize the titer of the imbibed blood. At 14 dpi, 32 live ZIKV exposed mosquitoes were placed in three different cages and allowed to feed on a mixture of Manuka honey, blue food dye and 1% ATP (100 mM) that was coated on Whatman filter paper cards (FP, 3 cards /cage) that were placed on the mesh at the top of the cage to collect mosquito saliva to assess transmission of ZIKV. At 16 dpi, 32 mosquito bodies (without legs) and the filter paper cards were stored individually in 1.5 ml tubes and immediately placed at −80°C for later determination of the infection rate in the mosquitoes and their ability to transmit ZIKV to the filter papers. This experiment was repeated except 34 blood engorged mosquitoes were held in screened cages and provided filter paper cards coated with honey from 14 to 16 dpi and plaque assay used to detect ZIKV on filter paper cards.

Quantitative RT- PCR to detect ZIKV RNA in blood and bodies was completed using the iTaq™ Universal Probes One-Step kit (BioRad, Hercules, CA) on the Bio-Rad CFX96™ Real-Time PCR Detection System. In the initial experiment, ZIKV was detected in saliva on filter paper cards using qRT-PCR as described above. The RNA was extracted from samples using Trizol reagent (Thermo Fisher, Waltham, MA). Specific ZIKV primers (IDT, Coralville, IA) were designed to the NS2B gene of a ZIKV isolate collected from human blood in Salvador, Brazil in 2015, (accession number KX520666). The primers and probe sequences are: REV: GGGAGAAATCACCACTCTCATC; FWD: GTTACGTGGTCTCAGGAAAGAG; PROBE: (5′ 6- FAM/ TGCGGAAGT/ZEN/CACTGGAAACAGTCC-3IABkFQ). ZIKV titers were calculated as described elsewhere (Smartt et al., 2017). A standard curve for ZIKV titer was obtained by serial dilution of a ZIKV stock [7.8 log10 ZIKV plaque-forming unit equivalents (pfue)/ml]. The standard curve was defined as the regression line of the logarithm of standard copy number versus quantification cycle (Cq) value. Titer difference between groups was determined using Kruskal Wallis –test (PRISM 7).

Plaque assays on Vero cells to estimate ZIKV titers in the saliva samples eluted from the filter paper cards during the second experiment were performed as described elsewhere (Kang et al., 2014). Briefly, the Vero cell line was maintained on Dulbecco's modified Eagle's medium (DMEM, Gibco) supplemented with 10% FBS (Sigma), 1% L-glutamine, and 1% penicillin-streptomycin (Invitrogen) at 37°C and 5% CO_2_. The filter paper cards that contained saliva were homogenized in DMEM with a Bullet Blender (NextAdvance), serially diluted, and then inoculated onto cells in 24 well plates. Plates were rocked for 15 min at room temperature, and then incubated for 45 min at 37°C and 5% CO_2_. Subsequently, 0.5 ml of DMEM containing 2% FBS and 0.8% methylcellulose were added to each well, and plates were incubated for 4 days at 37°C and 5% CO_2_. In order to count the plaque-forming units (pfu), plates were fixed and stained with methanol/acetone and 1% crystal violet mixture.

## Results

The blood fed to female *Cx. quinquefasciatus* contained 5.7 log 10 ZIKV pfue/ml, a titer that is lower than what has been used in some studies (Amraoui et al., 2016; Weger-Lucarelli et al., 2016), and the average ZIKV titer in freshly fed mosquitoes was 3.5 ± 0.1 log10 ZIKV pfue/ml. RNA from whole adult *Cx. quinquefasciatus* mosquito bodies collected 16 dpi after imbibing blood containing ZIKV were screened by qRT-PCR and revealed 9 female mosquito bodies with ZIKV RNA (Infection Rate = 28%, Table 1). The mean titer of ZIKV in the 9 bodies was 5.85 ± 5.8 log10 ZIKV pfue/ml (average ± SEM) and is not notably different from the titer in freshly fed mosquitoes. Analysis of RNA in saliva eluted from the filter paper at 16 dpi revealed an average titer of 5.6 ± 4.5 log10 ZIKV pfue/ml per card (Table 1) and there was no significant difference in the titers per cage (*P* > 0.1, *N* = 3).

**Table 1:** Detection of ZIKV by qRT-PCR in orally infected *Culex quinquefasciatus*.

**Cage Number**	**Number of positive mosquito bodies/cage**	**Average Cq/Body ± SEM**	**Average body titer ± SEM (PFUe/ml)log_10_**	**Number of filter papers (FP)**	**Average Cq FP ± SEM**	**Average FP titer ± SEM (PFUe/ml)log_10_**
1	2	27.1 ± 9.2	6.50 ± 6.5	3	26.1 ± 0.4	5.6 ± 4.9
2	4	35.0 ± 0.6	2.46 ± 1.9	3	26.1 ± 0.2	5.5 ± 4.6
3	3	37.1 ± 0.5	1.89 ± 1.3	3	25.6 ± 0.1	5.7 ± 4.6
Total	9	34.0 ± 6.1	5.85± 5.8	9	25.9 ± 0.2	5.6 ± 4.5

A follow up ZIKV infection experiment using the same methods and mosquitoes from the same population was conducted (Average titer of freshly fed females = 5.8 ± 0.101 log10 ZIKV pfue/ml). Quantitative RT-PCR to detect ZIKV RNA in the mosquitoes from the cages revealed positive bodies in cages 1 and 2 (Infection Rate = 55%). Plaque assays of the saliva samples eluted from the filter paper cards attached to cage 1 and 2 revealed the presence of live virus (Table 2). The average titer on the filter paper cards was 5.02 log 10 ZIKV pfu/ml.

**Table 2:** Detection of ZIKV in orally infected *Culex quinquefasciatus*.

**Cage number**	**Number of positive mosquito bodies/cage**	**Average Cq/Body± SEM**	**Average Body Titer ± SEM (PFUe/ml)log_10_**
**QUANTITATIVE RT-PCR DETECTION OF ZIKV**			
1	9	33.3 ± 0.3	2.89 ± 2.1
2	2	36.4 ± 1.8	2.25 ± 2.1
Total	11	33.8 ± 1.2	2.82 ± 2.1
**Cage Number**	**Total number of mosquitoes/cage**	**Titer (PFU/ml)log**_10_	
**PLAQUE ASSAY DETECTION OF ZIKV FROM SALIVA ON FILTER PAPER**			
1	10	5.32	
2	10	2.30	
Total	20	Average	5.02

## Discussion

Detection of ZIKV RNA in laboratory infected *Cx. quinquefasciatus* is not novel, however this report is the first laboratory study to show positive plaque assay results of live ZIKV collected from saliva from colonized *Cx. quinquefasciatus*. The results are proof of ZIKV transmission by this *Cx. quinquefasciatus* population under the conditions of the study. Though the average ZIKV RNA did not significantly increase in infected mosquitoes at 16 dpi compared to freshly-fed mosquitoes it is likely that what was detected at 16 dpi represented live ZIKV in at least some of the ZIKV positive mosquitoes. Since ZIKV RNA was not detected in all ZIKV exposed mosquitoes, it is unlikely that dead ZIKV or remnant ZIKV RNA would be able to infect salivary glands and resulting saliva after 16 dpi with no decline in titer. Although using a long established laboratory population may have contributed to their ability to transmit ZIKV, previous studies of ZIKV competence have also used established laboratory *Culex* populations with mixed results (Amraoui et al., 2016; Weger-Lucarelli et al., 2016; Guedes et al., 2017; Kenney et al., 2017). Laboratory reared *Cx. quinquefasciatus* from China infected with a contemporary isolate of ZIKV revealed ZIKV RNA in midgut tissues as early as 2 dpi and infected mosquitoes were allowed to feed on infant mice from which ZIKV RNA was later detected (Guo et al., 2016). During a ZIKV outbreak in Recife, Brazil, field collected *Cx. quinquefasciatus* were positive by qRT-PCR for ZIKV and mosquitoes from a colony of *Cx. quinquefasciatus* salivated ZIKV RNA, further validating its potential as a vector of ZIKV (Guedes et al., 2017). Field collections of mosquitoes from Mexico included *Aedes* and *Culex* species containing live ZIKV. The authors also revealed the presence of live virus in dissected salivary glands of wild mosquitoes from both species further supporting the need to study additional mosquito species for ZIKV vector competence (Elizondo-Quiroga et al., 2018). Live ZIKV was detected in the bodies of experimentally infected *Cx. quinquefasciatus* with infection rates of 17% at 14 dpi (Amraoui et al., 2016). Although this was lower than our observations their mosquitoes ingested a higher titer than what was provided in the present report. Another laboratory study showed that ZIKV infection of *Culex* species was virus lineage specific, refractory to infection under the conditions of the study though their *Cx. quinquefasciatus* population had been in the laboratory longer than the population used in our study (Kenney et al., 2017). Intrathoracic inoculation with both ZIKV lineages resulted in infected mosquito bodies without dissemination or transmission, suggesting a midgut infection barrier (Kenney et al., 2017). A *Cx. quinquefasciatus* population from Vero Beach, Florida, provided with a higher titer of ZIKV than used here, did not show the presence of ZIKV RNA in mosquito bodies (Huang et al., 2016) consistent with our view that our detection of ZIKV RNA post infection represent live ZIKV and not remnant ZIKV RNA from the blood meal.

The infection rates for *Cx. quinquefasciatus* we observed were lower compared to laboratory studies with *Ae. aegypti* and ZIKV (Chouin-Carneiro et al., 2016; Weger-Lucarelli et al., 2016). Analysis of the viral transmission potential based on RNA extracted from saliva on the filter paper cards revealed ZIKV RNA on each card consistent with the transmission of ZIKV by this species under the conditions of the experiment that supports there are likely specific populations of *Cx. quinquefasciatus* able to salivate ZIKV under unknown suitable conditions (Guo et al., 2016; Guedes et al., 2017). In our studies, the estimation of ZIKV RNA molecules in the saliva deposited on the filter papers at 16 dpi and detection of live virus in saliva on the cards by plaque assay is likely the result of several mosquitoes contributing to the saliva on the cards multiple times over 2 days of feeding, although in both cases the observed titers were also low. Caution is needed when making conclusions about the contribution of this mosquito species to future ZIKV cases until the competence for ZIKV by recently collected field populations of *Cx. quinquefasciatus* from the same region is evaluated under varying environmental conditions.

These findings support that there exists population-specific variability in the vector competence of *Cx. quinquefasciatus* for ZIKV like other mosquito-arboviral systems (Tabachnick, 2013). It affirms that there are biological and environmental conditions where *Cx. quinquefasciatus* mosquitoes are likely competent for ZIKV and these mosquitoes may play a role in ZIKV transmission to humans. Varying yet unknown environmental conditions likely influence variation in vector competence that has been reported in different studies that have resulted in different conclusions about the role of *Culex* in ZIKV transmission. We urge caution in making sweeping generalizations of ZIKV transmission capability. For example, further studies are warranted to ascertain those conditions that influence *Culex* vector competence for ZIKV in different localities where *Cx. quinquefasciatus* could be important in ZIKV epidemiology.

## Data availability statement

The raw data supporting the conclusions of this manuscript will be made available by the authors, without undue reservation, to any qualified researcher.

## Ethics statement

Chickens used to maintain mosquitoes complied with approved guidelines of the Institutional Animal Care and Use Committee (IACUC) at the University of Florida (IACUC Study #201507682).

## Author contributions

CS and DS: Conceived and designed the experiments; CS, DS, SK, and WT: Performed the experiments; DS and SK: Analyzed the data; CS, DS, and WT: Wrote the paper.

### Conflict of interest statement

The authors declare that the research was conducted in the absence of any commercial or financial relationships that could be construed as a potential conflict of interest.
